# Sodium fluoride induces renal inflammatory responses by activating NF-κB signaling pathway and reducing anti-inflammatory cytokine expression in mice

**DOI:** 10.18632/oncotarget.19006

**Published:** 2017-07-05

**Authors:** Qin Luo, Hengmin Cui, Huidan Deng, Ping Kuang, Huan Liu, Yujiao Lu, Jing Fang, Zhicai Zuo, Junliang Deng, Yinglun Li, Xun Wang, Ling Zhao

**Affiliations:** ^1^ College of Veterinary Medicine, Sichuan Agricultural University, Wenjiang, Chengdu, China; ^2^ Key Laboratory of Animal Diseases and Environmental Hazards of Sichuan Province, Sichuan Agriculture University, Wenjiang, Chengdu, China

**Keywords:** sodium fluoride, inflammatory responses, NF-κB, anti-inflammatory cytokines, kidney, Immunology and Microbiology Section, Immune response, Immunity

## Abstract

Fluoride is widely distributed in the environment and often results in adverse health effects on animals and human beings. It has been proved that fluoride can induce inflammatory responses *in vitro*. However, very limited reports are focused on fluoride-induced inflammatory responses *in vivo*. In this study, mice were used to investigate sodium fluoride (NaF) induced renal inflammatory responses and the potential mechanism by using the methods of pathology, biochemistry, enzyme-linked immunosorbent assay (ELISA), quantitative real-time polymerase chain reaction (qRT-PCR) and western blot. A total of 240 ICR mice were randomly divided into four equal groups: the control group and three experimental groups (NaF was given orally at the dose of 0, 12, 24 and 48 mg/kg body weight for 42 days, respectively). The results showed that NaF in excess of 12 mg/kg induced the renal histopathological lesions, and inflammatory responses *via* the activation of nuclear factor-kappa B (NF-κB) signaling pathway and the reduction of anti-inflammatory cytokines expression. The activation of NF-κB signaling pathway was characterized by increasing the nitric oxide (NO) and prostaglandin E_2_ (PGE_2_) contents, inducible nitric oxide synthase (iNOS) activities and mRNA expression levels, and the mRNA and protein expression levels of cyclooxygenase-2 (COX-2), tumor necrosis factor-α (TNF-α), interleukin-1β (IL-1β), interleukin-6 (IL-6) and interleukin-8 (IL-8) in three NaF-treated groups. Concurrently, the mRNA and protein expression levels of the anti-inflammatory cytokines including interleukin-4 (IL-4) and interleukin-10 (IL-10) were decreased in three experimental groups when compared with those in the control group.

## INTRODUCTION

Fluoride in small quantity is necessary for normal formation of dental enamel and mineralization of bones [1, 2]. However, its excessive ingestion may lead to damages and pathological changes in many tissues and organs including kidney [3–26]. Functional disorder of the kidney has been considered as a consequence of fluorosis [27, 28] though its pathogenesis is not well understood at present.

For organisms, inflammation is not only a defensive response to resist the harmful stimulus, but also a healing process for repairing injured tissue [29, 30]. It is well known that inflammation has many beneficial effects on animals and human beings. However, chronic or sustained inflammation is detrimental [31]. Inflammation can be triggered by a variety of inducers such as pathogens, damaged cells, or toxic compounds [20]. Previous studies have demonstrated that fluoride-caused testicular toxicity is associated with the inflammatory responses in mice [20, 21]. Gutowska et al. [32] has shown that even low levels of fluoride in differentiated human THP1 monocytes/macrophages may alter activities of cyclooxygenase-1 (COX-1) and cyclooxygenase-2 (COX-2), which involved in the initiation and development of inflammation. Ridley et al. [33] has also found that acute inflammatory responses may contribute to the subsequent development of fluoride-induced airway diseases. As the primary organ associated with excretion and retention of fluoride, kidney is very sensitive to the toxicity of fluoride [34]. However, there are no studies about fluoride-induced inflammatory responses in the kidney of animals and human beings up to now.

Nuclear factor-kappa B (NF-κB), as a transcription factor, involves in the inflammatory and immune responses *via* its ability to induce the expression of its downstream genes [35]. Some of NF-κB's downstream genes, including inducible nitric oxide synthase (iNOS), COX-2, tumor necrosis factor-α (TNF-α) and interleukin-1β (IL-1β), are closely correlated to inflammatory responses [1]. Tian et al. [36] has demonstrated that fluoride can regulate the mRNA expression of NF-κB, iNOS, TNF-α and IL-1β in mouse peritoneal macrophages. Yan et al. [37] has also reported that fluoride can induce the expression of TNF-α, IL-1β and interleukin-6 (IL-6) in rat brain. In addition, De la Fuente et al. [38] has found that low levels of fluoride can decrease the expression of the anti-inflammatory cytokine interleukin-10 (IL-10) in murine macrophages. However, the effects of fluoride on NF-κB activation and inflammatory mediators in the kidney of animals and human beings are unclearly at present.

In the present study, mice were used to explore how sodium fluoride (NaF) induced renal inflammatory responses by measuring nitric oxide (NO) and prostaglandin E_2_ (PGE_2_) contents, iNOS activities and mRNA expression levels, NF-κB mRNA expression levels, p-NF-κB protein expression levels, as well as inhibitors of kappa B (IκB), COX-2, TNF-α, IL-1β, IL-6, interleukin-8 (IL-8), interleukin-4 (IL-4), IL-10 mRNA and protein expression levels with the methods of pathology, biochemistry, enzyme-linked immunosorbent assay (ELISA), quantitative real-time polymerase chain reaction (qRT-PCR) and western blot.

## RESULTS

### Histopathological changes in the kidney

**Figure 1:**
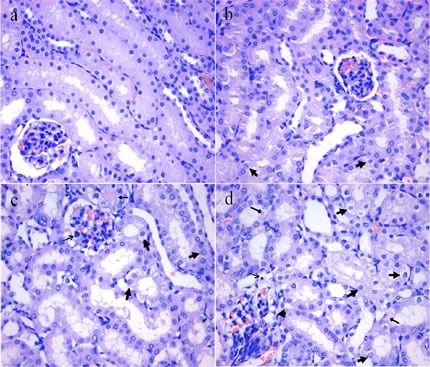
Histopathological changes in the kidney at 21 days of the experiment **a.** The control group (H&E × 400). **b.** The 12 mg/kg group. Tubular cells show slightly granular and vacuolar degeneration (, H&E × 400). **c.** The 24 mg/kg group. Tubular cells show granular and vacuolar degeneration ( ). The infiltration of inflammatory cells ( ) are observed (H&E × 400). **d.** The 48 mg/kg group. Tubular cells show marked granular and vacuolar degeneration ( ). Also, necrotic tubular cells ( ), infiltration of inflammatory cells ( ) and swelled glomeruli without capsular space ( ) are observed (H&E × 400).

**Figure 2:**
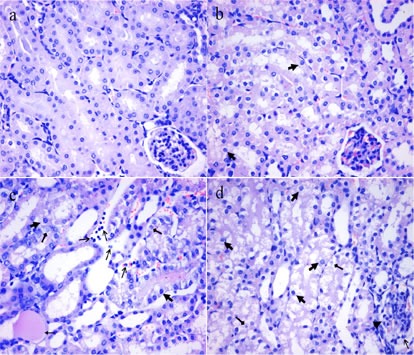
Histopathological changes in the kidney of mice at 42 days of the experiment **a.** The control group (H&E × 400). **b.** The 12 mg/kg group. Tubular cells show granular and vacuolar degeneration (, H&E × 400). **c.** The 24 mg/kg group. Tubular cells show marked granular and vacuolar degeneration ( ). Necrotic tubular cells ( ), renal tubular hyaline casts ( ) and infiltration of inflammatory cells ( ) are observed (H&E × 400). **d.** The 48 mg/kg group. Tubular cells show marked granular and vacuolar degeneration ( ). Also, necrotic tubular cells ( ), infiltration of inflammatory cells ( ) and swelling glomeruli without capsular space ( ) are observed (H&E × 400).

Figures 1 and 2 showed that the degeneration and necrosis of the tubular cells, infiltration of inflammatory cells, swelled glomeruli as well as the renal tubular hyaline casts were observed in the NaF-treated groups. Also, the histopathological lesions induced by fluoride were changed in a dose- and time-dependent manner. The above lesions were not observed in the control group.

### Changes of mRNA and protein expression levels of NF-κB and IκB in the kidney

**Figure 3:**
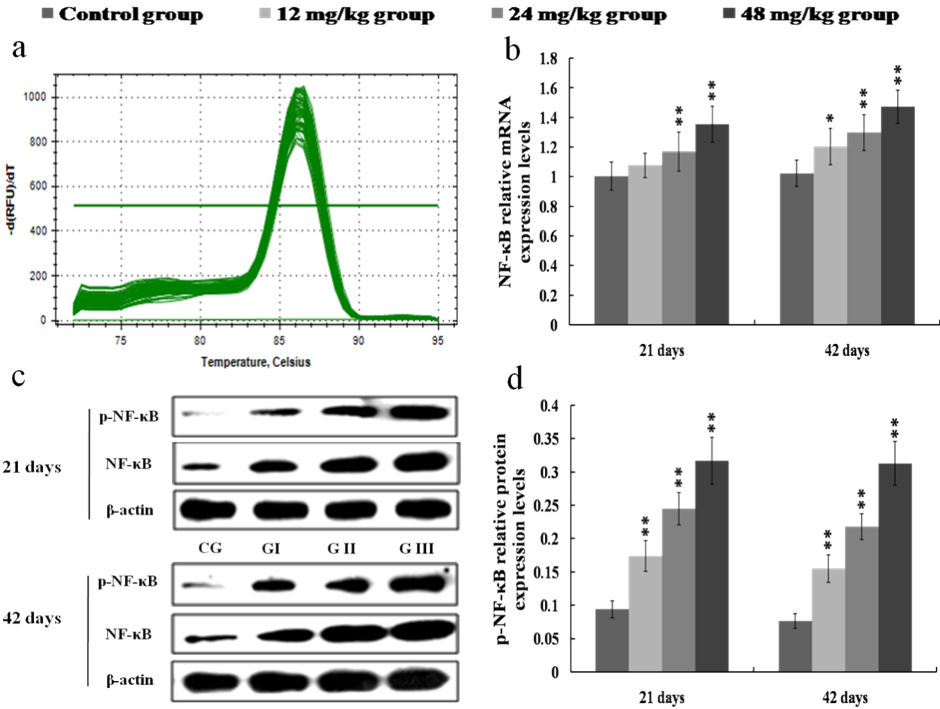
Changes of mRNA and protein expression levels of NF-κB in the kidney at 21 and 42 days of the experiment **a.** The melting curve analysis of NF-κB. **b.** The relative mRNA expression levels of NF-κB. **c.** The western blot assay of NF-κB and p-NF-κB. **d.** The relative protein expression levels of p-NF-κB. CG: Control group; GI: 12mg/kg group; GII: 24mg/kg group; GIII: 48mg/kg group. Data are presented with the mean ± standard deviation (*n* = 8), *p < 0.05, compared with the control group; **p < 0.01, compared with the control group.

**Figure 4:**
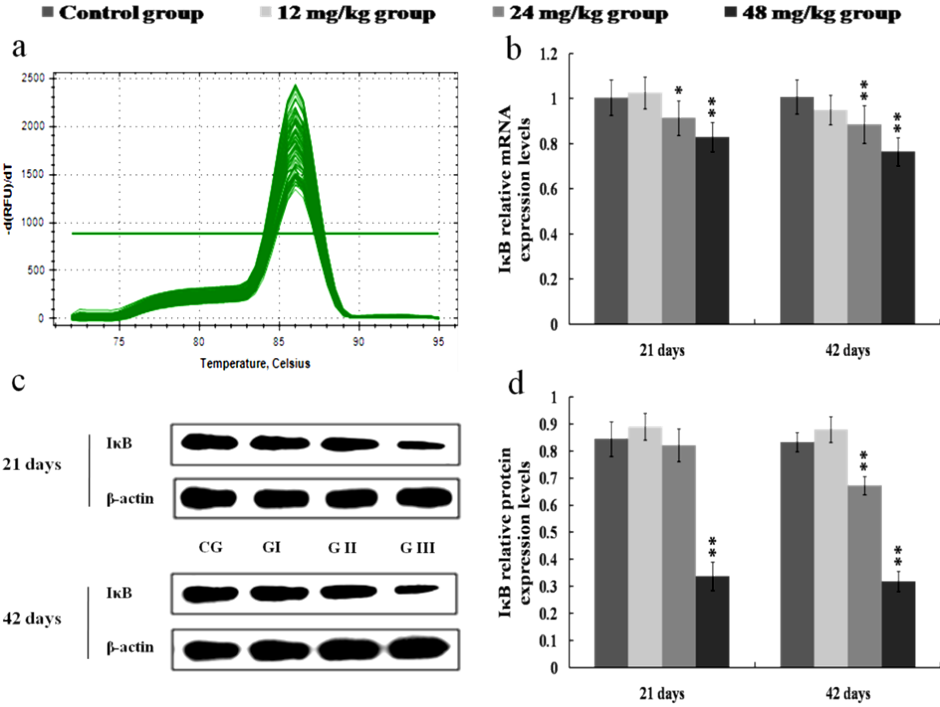
Changes of mRNA and protein expression levels of IκB in the kidney at 21 and 42 days of the experiment **a.** The melting curve analysis of IκB. **b.** The relative mRNA expression levels of IκB. **c.** The western blot assay of IκB. **d.** The relative protein expression levels of IκB. CG: Control group; GI: 12mg/kg group; GII: 24mg/kg group; GIII: 48mg/kg group. Data are presented with the mean ± standard deviation (*n* = 8), *p < 0.05, compared with the control group; **p < 0.01, compared with the control group.

The NF-κB mRNA expression levels were increased (*p* < 0.05) in the 12 mg/kg group at 42 days of the experiment and were markedly increased (*p* < 0.01) in the 24 and 48 mg/kg groups at 21 and 42 days of the experiment in comparison with those in the control group, as shown in Figures 3a-3b. Figures 3c-3d showed that p-NF-κB protein expression levels were significantly higher (*p* < 0.01) in the 12, 24 and 48 mg/kg groups at 21 and 42 days of the experiment than those in the control group. The IκB mRNA and protein expression levels were lower (*p* < 0.01 or *p* < 0.05) in the 24 and 48 mg/kg groups at 21 and 42 days of the experiment than those in the control group. The results were shown in Figure 4.

### Changes of NO contents as well as iNOS activities and mRNA expression levels in the kidney

**Figure 5:**
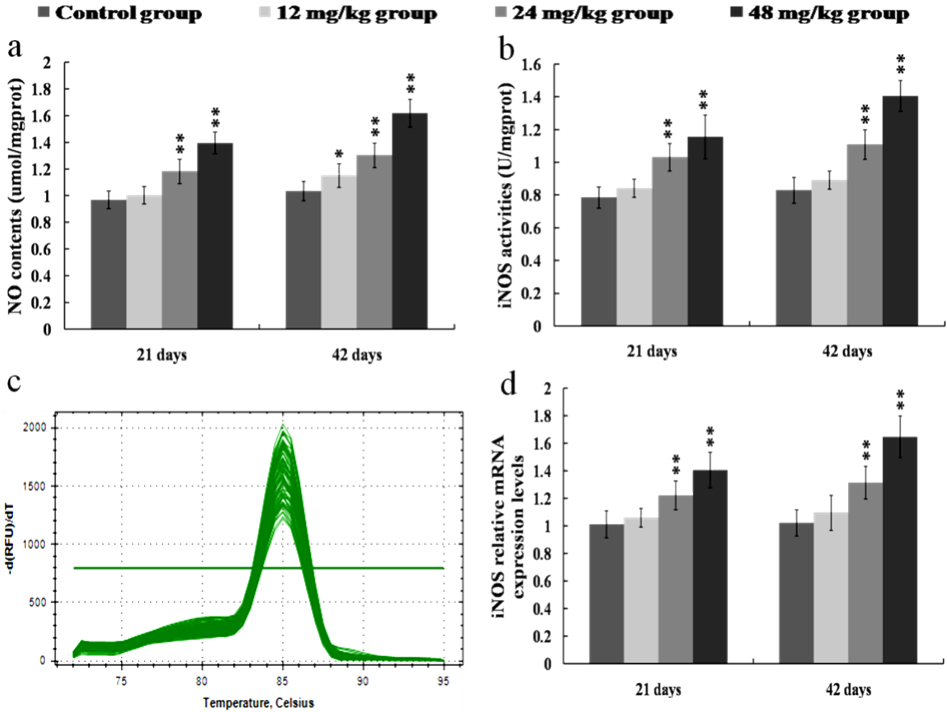
Changes of NO contents as well as iNOS activities and mRNA expression levels in the kidney at 21 and 42 days of the experiment **a.** The NO contents in the kidney. **b.** The iNOS activities in the kidney. **c.** The melting curve analysis of iNOS. **d.** The relative mRNA expression levels of iNOS. CG: Control group; GI: 12mg/kg group; GII: 24mg/kg group; GIII: 48mg/kg group. Data are presented with the mean ± standard deviation (*n* = 8). *p < 0.05, compared with the control group; **p < 0.01, compared with the control group.

Figure 5a showed that NO contents were enhanced (*p* < 0.05) in the 12 mg/kg groups at 42 days of the experiment and were significantly enhanced (*p* < 0.01) in the 24 and 48 mg/kg groups at 21 and 42 days of the experiment when compared with those in the control group. The activities and mRNA expression levels of iNOS were higher (*p* < 0.01) in the 24 and 48 mg/kg groups at 21 and 42 days of the experiment than those in the control group, as shown in Figures 5b-5d.

### Changes of PGE_2_ contents as well as COX-2 mRNA and protein expression levels in the kidney

**Figure 6:**
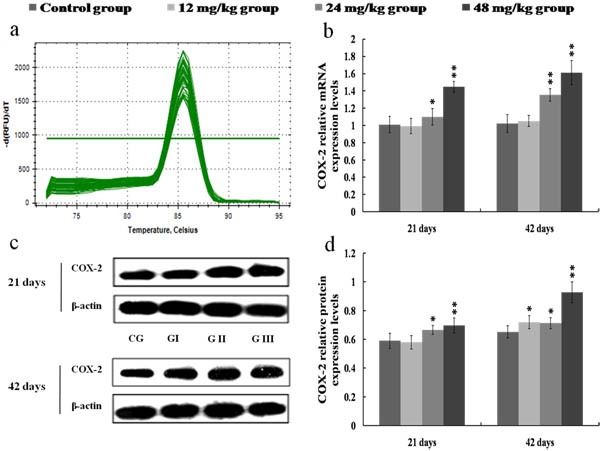
Changes of mRNA and protein expression levels of COX-2 in the kidney at 21 and 42 days of the experiment **a.** The melting curve analysis of COX-2. **b.** The relative mRNA expression levels of COX-2. **c.** The western blot assay of COX-2. **d.** The relative protein expression levels of COX-2. CG: Control group; GI: 12mg/kg group; GII: 24mg/kg group; GIII: 48mg/kg group. Data are presented with the mean ± standard deviation (*n* = 8). *p < 0.05, compared with the control group; **p < 0.01, compared with the control group.

**Figure 7:**
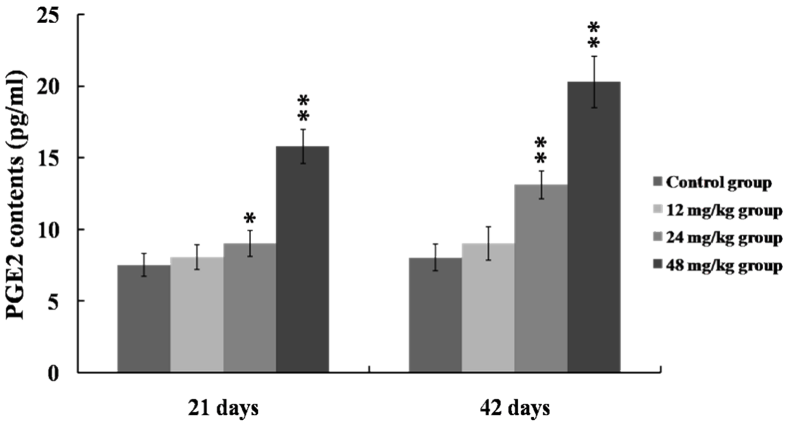
Changes of PGE_2_ contents in the kidney at 21 and 42 days of the experiment Data are presented with the mean ± standard deviation (*n* = 8). *p < 0.05, compared with the control group; **p < 0.01, compared with the control group.

As shown in Figure 6, the COX-2 mRNA and protein expression levels were significantly higher (*p* < 0.01 or *p* < 0.05) in the 24 and 48 mg/kg groups at 21 and 42 days of the experiment than those in the control group. Figure 7 showed that PGE_2_ contents were markedly increased (*p* < 0.01 or *p* < 0.05) in the 24 and 48 mg/kg groups at 21 and 42 days of the experiment in comparison with those in the control group.

### Changes of pro-inflammatory cytokines mRNA and protein expression levels in the kidney

**Figure 8:**
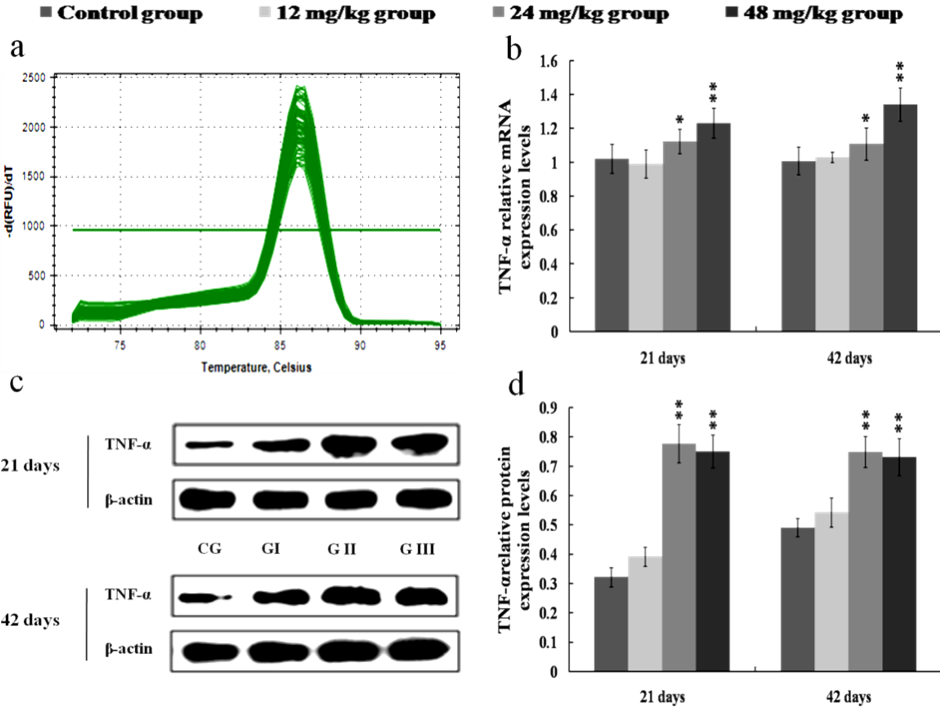
Changes of mRNA and protein expression levels of TNF-α in the kidney at 21 and 42 days of the experiment **a.** The melting curve analysis of TNF-α. **b.** The relative mRNA expression levels of TNF-α. **c.** The western blot assay of TNF-α. **d.** The relative protein expression levels of TNF-α. CG: Control group; GI: 12mg/kg group; GII: 24mg/kg group; GIII: 48mg/kg group. Data are presented with the mean ± standard deviation (*n* = 8). *p < 0.05, compared with the control group; **p < 0.01, compared with the control group.

**Figure 9:**
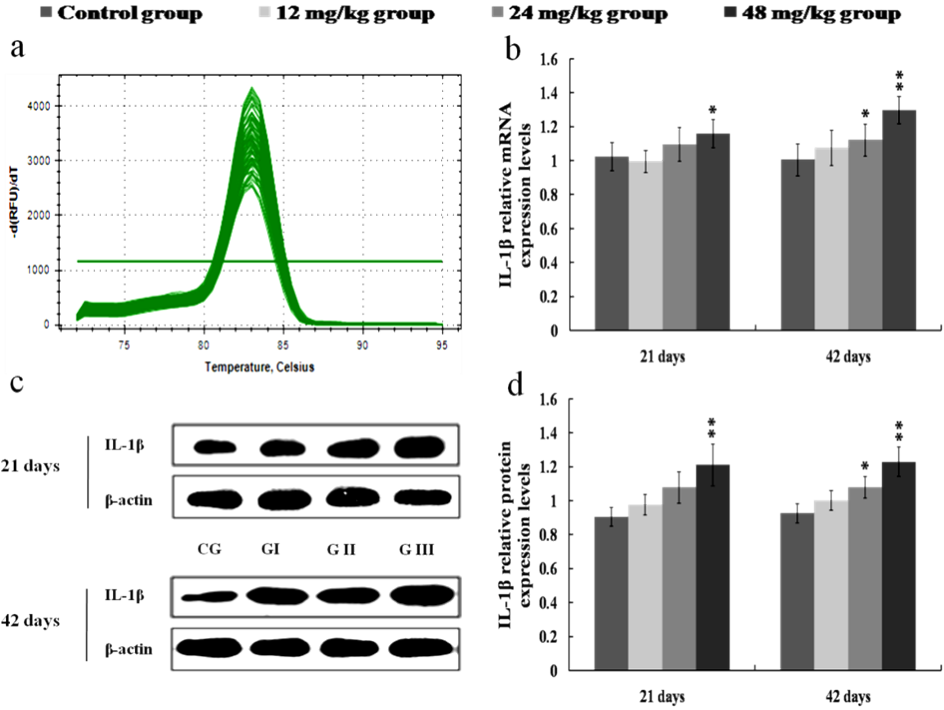
Changes of mRNA and protein expression levels of IL-1β in the kidney at 21 and 42 days of the experiment **a.** The melting curve analysis of IL-1β. **b.** The relative mRNA expression levels of IL-1β. **c.** The western blot assay of IL-1β. **d.** The relative protein expression levels of IL-1β. CG: Control group; GI: 12mg/kg group; GII: 24mg/kg group; GIII: 48mg/kg group. Data are presented with the mean ± standard deviation (*n* = 8). *p < 0.05, compared with the control group; **p < 0.01, compared with the control group.

**Figure 10:**
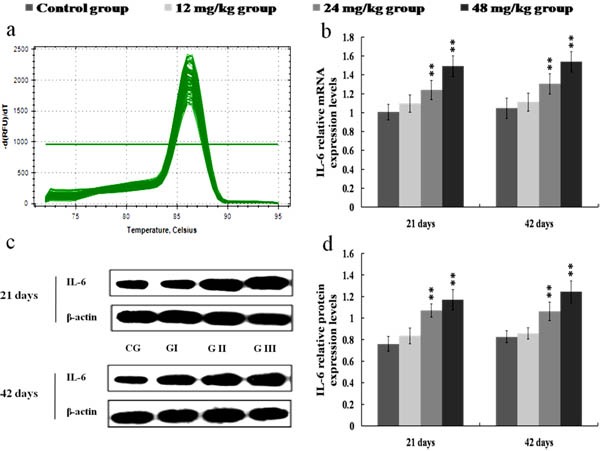
Changes of mRNA and protein expression levels of IL-6 in the kidney at 21 and 42 days of the experiment **a.** The melting curve analysis of IL-6. **b.** The relative mRNA expression levels of IL-6. **c.** The western blot assay of IL-6. **d.** The relative protein expression levels of IL-6. CG: Control group; GI: 12mg/kg group; GII: 24mg/kg group; GIII: 48mg/kg group. Data are presented with the mean ± standard deviation (*n* = 8). *p < 0.05, compared with the control group; **p < 0.01, compared with the control group.

**Figure 11:**
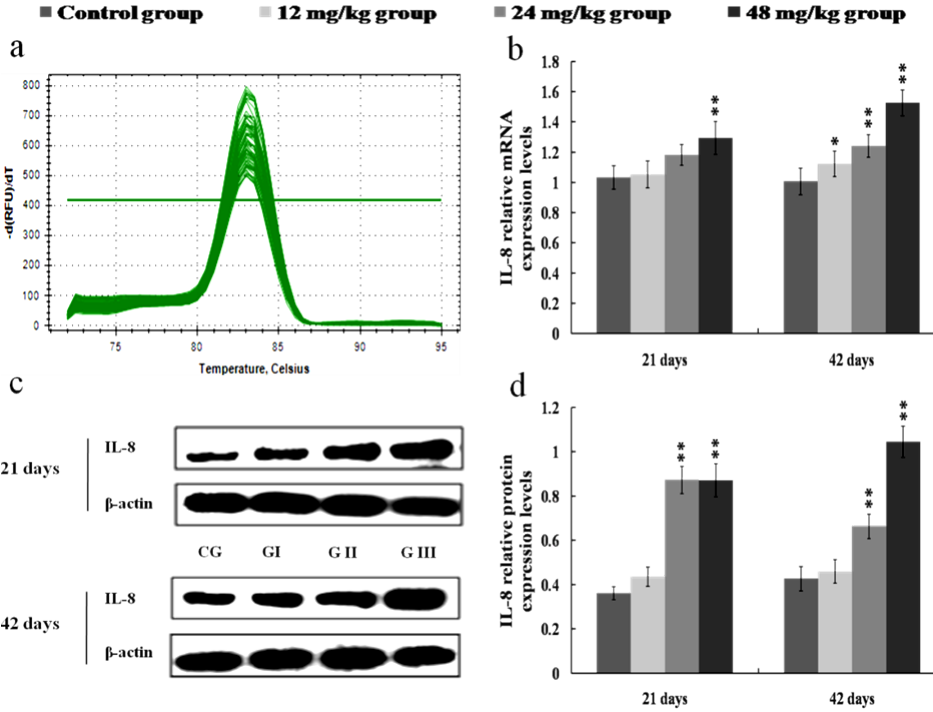
Changes of mRNA and protein expression levels of IL-8 in the kidney at 21 and 42 days of the experiment **a.** The melting curve analysis of IL-8. **b.** The relative mRNA expression levels of IL-8. **c.** The western blot assay of IL-8. **d.** The relative protein expression levels of IL-8. CG: Control group; GI: 12mg/kg group; GII: 24mg/kg group; GIII: 48mg/kg group. Data are presented with the mean ± standard deviation (*n* = 8). *p < 0.05, compared with the control group; **p < 0.01, compared with the control group.

The results in Figures 8, 9, 10 and 11 showed that the mRNA and protein expression levels of TNF-α, IL-1β, IL-6 and IL-8 were markedly increased (*p* < 0.01 or *p* < 0.05) in the 24 and 48 mg/kg groups from 21 to 42 days of the experiment when compared with those in the control group.

### Changes of anti-inflammatory cytokines mRNA and protein expression levels in the kidney

**Figure 12:**
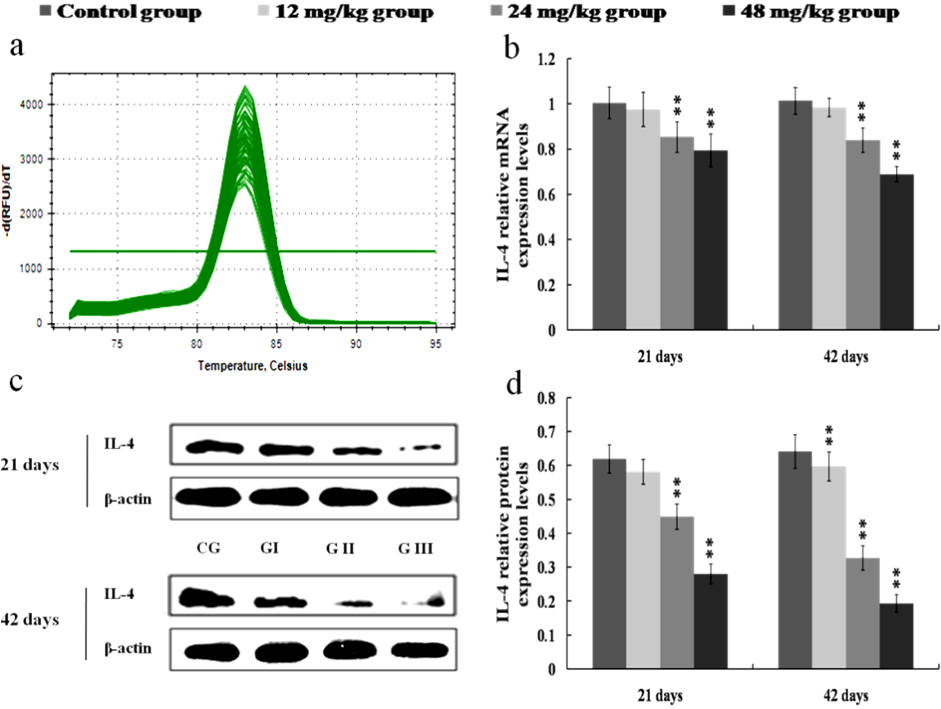
Changes of mRNA and protein expression levels of IL-4 in the kidney at 21 and 42 days of the experiment **a.** The melting curve analysis of IL-4. **b.** The relative mRNA expression levels of IL-4. **c.** The western blot assay of IL-4. **d.** The relative protein expression levels of IL-4. CG: Control group; GI: 12mg/kg group; GII: 24mg/kg group; GIII: 48mg/kg group. Data are presented with the mean ± standard deviation (*n* = 8). *p < 0.05, compared with the control group; **p < 0.01, compared with the control group.

**Figure 13:**
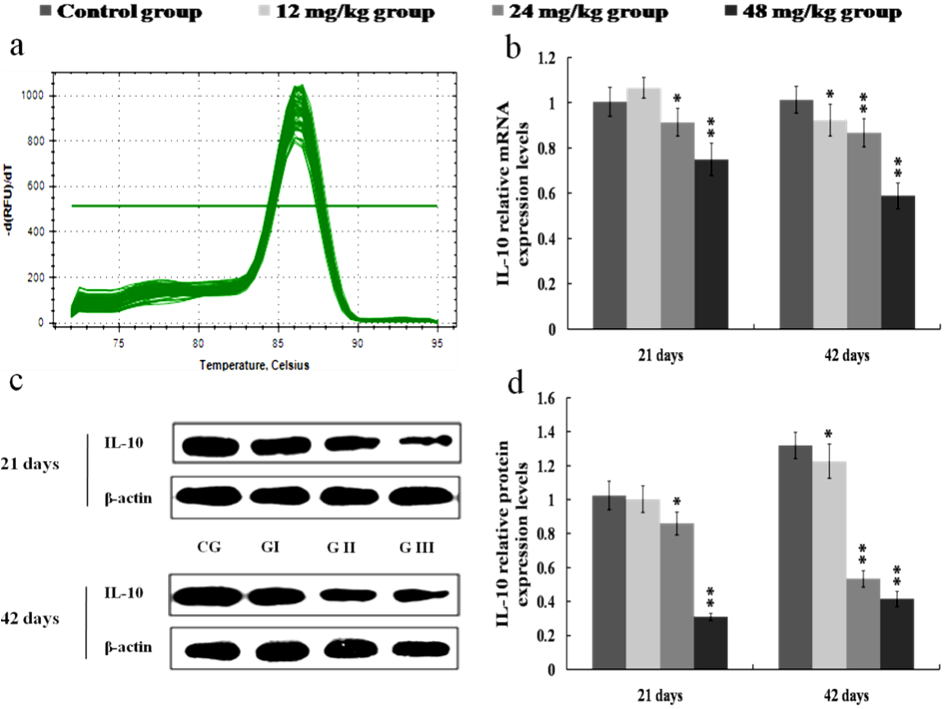
Changes of mRNA and protein expression levels of IL-10 in the kidney at 21 and 42 days of the experiment **a.** The melting curve analysis of IL-10. **b.** The relative mRNA expression levels of IL-10. **c.** The western blot assay of IL-10. **d.** The relative protein expression levels of IL-10. CG: Control group; GI: 12mg/kg group; GII: 24mg/kg group; GIII: 48mg/kg group. Data are presented with the mean ± standard deviation (*n* = 8). *p < 0.05, compared with the control group; **p < 0.01, compared with the control group.

The mRNA and protein expression levels of IL-4 and IL-10 were significantly reduced (*p* < 0.01 or *p* < 0.05) in the 24 and 48 mg/kg groups at 21 and 42 days of the experiment when compared with those in the control group. Also, the protein expression levels of IL-4 and IL-10 were lower (*p* < 0.01 or *p* < 0.05) in the 12 mg/kg groups at 42 days of the experiment than those in the control group. The results were shown in Figures 12 and 13.

## DISCUSSION

Exposure to fluoride has been shown to induce inflammatory responses in lung epithelial cells [39], HeLa cells [40], THP1 differentiated monocytes/macrophages [32], peritoneal macrophages [36], aorta [16], neuron [37] and testicle [20, 21]. However, very limited reports are focused on fluoride-induced inflammatory responses *in vivo*. There are no studies about the effects of fluoride on renal inflammatory responses in animals and human beings at present. The aim of this study was to define the fluoride-induced inflammatory responses in the kidney. Indeed, we found considerable evidence that NaF in excess of 12 mg/kg induced the renal inflammatory responses in mice, which was supported by the findings: activation of NF-κB signaling pathway and reduction of anti-inflammatory cytokines expression.

NF-κB is a transcription factor that can integrate a complex network of extracellular perturbations and intracellular signaling pathways, leading to the transcriptional regulation of a wide variety of genes correlated to inflammation [41]. Under inactive state, NF-κB exists in the cytoplasm, and is combined with its regulatory proteins called IκB [36]. When the IκB proteins are activated by some stimulus such as toxic compounds, pathogens, or pro-inflammatory cytokines, they are phosphorylated, ubiquitinated and subsequently degraded [42]. After IκB proteins degraded, NF-κB is dissociated from the cytoplasmic NF-κB-IκB complex, and translocates to the nucleus. Then NF-κB binds to its cognate DNA binding sites to regulate the expression of corresponding pro-inflammatory genes [43]. In this study, the IκB mRNA and protein expression levels were reduced, the NF-κB mRNA expression levels and p-NF-κB protein expression levels were increased in the 24 and 48 mg/kg groups, demonstrating that NaF-induced IκB degradation promoted the activation and translocation of NF-κB.

Once NF-κB is activated, NF-κB then regulates the expression of a series of pro-inflammatory mediators, including iNOS, COX-2, TNF-α, IL-1β, IL-6 and IL-8 [43, 44]. Up-regulation of iNOS occurs mostly in association with the inflammation and infection as a part of defensive reaction [36]. Meanwhile, iNOS can catalyze the production of NO, and NO is known to participate in the inflammation responses by increasing the inflammatory cell infiltration and vascular permeability [20, 45, 46]. In the present study, we found that the NO contents as well as the iNOS activities and mRNA expression levels were significantly increased in the 24 and 48 mg/kg groups, suggesting that NaF exposure induced the production of NO and iNOS, and that the up-regulation in the transcriptional expression of iNOS was the possible reason of the increase in NO generation. COX-2, as an important inflammatory mediator, is a major contributor to the production of PGE_2_. PGE_2_ can recruit the inflammatory cells and accelerate the process of peripheral inflammation induced by noxious stimulus [47]. In this study, the mRNA and protein expression levels of COX-2 were increased in the 24 and 48 mg/kg groups, and the PGE_2_ contents were also enhanced in the 24 and 48 mg/kg groups, which suggested that NaF induced the production of COX-2 and PGE_2_, and the PGE_2_ production in response to NaF exposure in the kidney was dependent on the up-regulation of COX-2 expression. Also, our results are consistent with the report that fluoride can increase the COX-2 expression and PGE_2_ production in A549 human pulmonary epithelial cells [33]. Other pro-inflammatory cytokines such as TNF-α, IL-1β and IL-6 have been shown to play an important role in cell signaling and systemic inflammation [48]. These cytokines are predominantly produced by activated monocytes/macrophages and T cells, and are involved in the up-regulation of inflammatory responses [18, 49]. Moreover, the cytokine IL-8 can recruit neutrophils and lymphocytes to the sites of infection or injury in inflammation responses [39]. In the present study, the mRNA and protein expression levels of pro-inflammatory cytokines including TNF-α, IL-1β, IL-6 and IL-8 were markedly increased in the 24 and 48 mg/kg groups. The above-mentioned results demonstrated that NaF in excess of 12 mg/kg can activate the NF-κB signaling pathway, and the activation of NF-κB signaling pathway is one of the pathways of NaF-induced renal inflammatory responses in mice.

As main anti-inflammatory cytokines, IL-4 and IL-10 are supposed to inhibit the detrimental effects of local pro-inflammatory responses [50, 51]. In this study, the mRNA and protein expression levels of IL-4 and IL-10 were reduced in the 12, 24 and 48 mg/kg groups, which contribute to occurrence of the renal inflammatory responses. And the reduction of IL-4 and IL-10 mRNA and protein expression levels may weakens their inhibiting effects on the production of pro-inflammatory mediators. The results are also consistent with the our previous reports that fluoride can decrease IL-4 contents in broiler's serum [52], cecal tonsil [53] and intestinal mucosa [18].

Imbalance between pro-inflammatory and anti-inflammatory mediators induces the renal inflammatory responses, which can result in renal histopathological lesions. Indeed, our histopathological results also showed that NaF-induced the degeneration and necrosis of the tubular cells, inflammatory cell infiltration, swelled glomeruli as well as the renal tubular hyaline casts in a dose- and time-dependent manner from 21 to 42 days during the experiment.

## CONCLUSIONS

**Figure 14:**
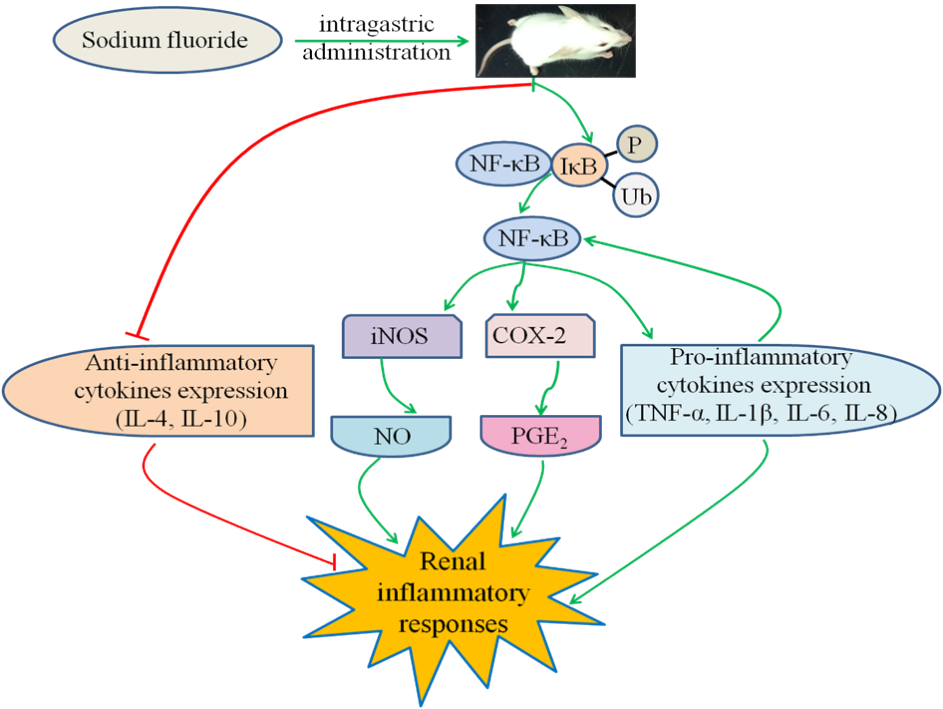
Schematic of NaF-induced the renal inflammatory responses in mice NaF can induce the renal inflammatory responses by increasing the NO and PGE_2_ contents, iNOS activities and mRNA expression levels as well as the COX-2, TNF-α, IL-1β, IL-6 and IL-8 mRNA and protein expression levels via the activation of NF-κB signaling pathway, and reducing the mRNA and protein expression levels of IL-4 and IL-10. The activation of NF-κB signaling pathway and reduction of anti-inflammatory mediator expression are the potential mechanisms of NaF-induced renal inflammatory responses.

The present study finds that NaF in excess of 12 mg/kg can induce the renal histological lesions, and inflammatory responses by increasing the NO and PGE_2_ contents, iNOS activities and mRNA expression levels as well as the COX-2, TNF-α, IL-1β, IL-6 and IL-8 mRNA and protein expression levels *via* the activation of NF-κB signaling pathway, and reducing the mRNA and protein expression levels of IL-4 and IL-10. The activation of NF-κB signaling pathway and reduction of anti-inflammatory mediator expression are the potential mechanisms of NaF-induced renal inflammatory responses, as shown in Figure 14.

## MATERIALS AND METHODS

### Chemicals and sources

NaF was purchased from Chengdu Kelong Chemical Co., Ltd. (Chengdu, China). Reagent kits for determination of biochemical parameters were purchased from Nanjing Jiancheng Bioengineering Institute of China (Nanjing, China). RNAiso Plus, Prim-Script^TM^ RT reagent Kit and SYBR^®^ Premix Ex Taq^TM^ II were obtained from Takara Biotechnology (Dalian) Co., Ltd. (Dalian, Liaoning, China). Radio-immunoprecipitation Assay (RIPA) lysis buffer (P0013C) and bicinchoninic acid (BCA) Protein Assay Kit (P0012) were purchased from Beyotime Biotechnology, China. RPMI 1640 (11875119) was supplied by Gibco, UK. The mouse NF-κB (ab32536), IκB (ab32518) and COX-2 (ab179800) were obtained from Abcam, UK. IL-6 (12912), p-NF-κB (3033), and anti-rabbit IgG (7074P2) were purchased from Cell Signaling Technology, USA. TNF-α (sc-1351) and IL-10 (sc-1783) were supplied by Santa Cruz Biotechnology, Inc. (Santa Cruz, CA, USA). IL-1β (abs115412), IL-4 (abs116760) and bicinchoninic acid (BCA) were obtained from Absin Bioscience Inc. (Absin, Shanghai, China). Anti-goat IgG antibody (BL004A) was supplied by Biosharp Bioscience Inc. (Biosharp, Anhui, China). Other chemicals including 75% ethanol, 100% ethanol, isopropyl alcohol and chloroform used in the experiment were of analytical grade.

### Experimental animals and treatment

A total of 240 four-week-old ICR mice obtained from the Chengdu Dossy Experimental Animals Co., Ltd. [License No. SCXK (Sichuan) 2008-24] were used in the present study. The animals were housed in separate polypropylene cages, and kept under the standard laboratory conditions (at a temperature of 20-22°C with a 12 h light/12 h dark photoperiod and 55-60% humidity). Diet and water were provided *ad libitum* throughout the experimental period.

After one week of acclimation, animals were randomly divided into four equal groups with 60 mice in each. Control group was given orally distilled water only and served as untreated group. Groups I, II and III were given orally NaF at the dose of 12, 24 and 48 mg/kg body weight, respectively. Mice were administered their respective doses daily by gavages for consecutively 42 days, and the gavage volume was 1ml/100g body weight.

All experimental procedures involving the use of mice were approved by the Animal Care and Use Committee, Sichuan Agricultural University.

### Histopathological observation

At 21 and 42 days of the experiment, kidneys of eight mice in each group were removed, fixed in 4% paraformaldehyde, dehydrated with increasing concentrations of ethanol, cleared with xylene and embedded in paraffin. And then kidneys were serial sectioned at 5 μm thickness, stained with hematoxylin and eosin (H&E), and observed by light microscopy.

### Determination of NO contents and iNOS activities in the kidney

At 21 and 42 days of the experiment, eight mice in each group were sacrificed and kidneys were removed immediately. Kidneys were washed using chilled saline solution, weighed, homogenized in nine volumes of ice-cold 0.9% NaCl solution and centrifuged at 3500 rpm for 10 min at 4°C. Then the supernatant was collected for the determination of NO contents and iNOS activities by biochemical methods according to the instructions of the reagent kits (NO, A013-2; iNOS, A014-1; Nanjing Jiancheng, China).

### Determination of PGE_2_ contents in the kidney by ELISA

At 21 and 42 days of the experiment, kidneys of eight mice in each group were taken to measure the PGE_2_ contents. Kidneys were weighed and homogenized in nine volumes of ice-cold 0.9% NaCl solution in a chilled homogenizer, and immediately centrifuged at 3500 rpm for 10 min at 4°C. Then the supernatant was immediately assayed for the PGE_2_ contents by ELISA as described by Gaca et al. [54]. The detected PGE_2_ contents were determined by the standard curve and were expressed as microgram per milliliter (pg/ml).

### Determination of inflammatory mediator mRNA expression levels in the kidney by qRT-PCR

At 21 and 42 days of the experiment, kidneys of eight mice in each group were removed, stored in liquid nitrogen, and then homogenized with liquid nitrogen for RNA extraction. The methods of RNA extraction and qRT-PCR analysis were same as the described by Guo et al. [55]. Briefly, the total RNA of the kidneys were extracted using RNAiso Plus (9109; Takara, China) following the manufacturer's instructions. The cDNA, used as the template for qRT-PCR analysis, was synthesized using a Prim-Script^TM^ RT reagent Kit (RR047A, Takara, China) following the manufacturer's instructions. Specific primers for the genes were designed and synthesized by Sangon (Shanghai, China) according to the Mus musculus sequences, and the expression levels of NF-κB, IκB, iNOS, COX-2, TNF-α, IL-1β, IL-6, IL-8, IL-4 and IL-10 were normalized to the expression levels of a house keeping gene, β-actin (Table 1).

**Table 1:** Primer sequences of genes selected for analysis by qRT-PCR

Target gene	Accession number	Primer	Primer sequence (5′-3′)	Product size	Tm (°C)
NF-κB	NM_008689	Forward	GTAACAGCAGGACCCAAGGA	121bp	59
		Reverse	AGCCCCTAATACACGCCTCT		
IκB	NM_010908	Forward	TGAGGACGAGGACGATAAGC	159bp	60
		Reverse	TCAGGAAGAGGTTTGGATGC		
iNOS	NM_010927	Forward	GAATCTTGGAGCGAGTTGTGG	139bp	60
		Reverse	AGGAAGTAGGTGAGGGCTTGG		
COX-2	NM_011198	Forward	GCCTGGTCTGATGATGTATGC	124bp	61
		Reverse	GAGTATGAGTCTGCTGGTTTGG		
TNF-α	NM_013693	Forward	CACGTCGTAGCAAACCACC	88bp	59
		Reverse	TGAGATCCATGCCGTTGGC		
IL-1β	NM_008361	Forward	AATGCCACCTTTTGACAGTGAT	132bp	61
		Reverse	TGCTGCGAGATTTGAAGCTG		
IL-6	NM_031168	Forward	AGGATACCACTCCCAACAGACC	141bp	60
		Reverse	AAGTGCATCATCGTTGTTCATACA		
IL-8	NM_011339	Forward	TTTCCACCGGCAATGAAG	115bp	59
		Reverse	TAGAGGTCTCCCGAATTGGA		
IL-4	NM_021283	Forward	GTCATCCTGCTCTTCTTTCTCG	115bp	61
		Reverse	ATGGCGTCCCTTCTCCTGT		
IL-10	NM_010548	Forward	GACAACATACTGCTAACCGACT	252bp	59
		Reverse	ATCACTCTTCACCTGCTCCAC		
β-actin	NM_007393	Forward	GCTGTGCTATGTTGCTCTAG	117bp	59
		Reverse	CGCTCGTTGCCAATAGTG		

qRT-PCR was performed on a Thermal Cycler (C1000, BIO RAD, USA) using SYBR^®^ Premix Ex Taq^TM^ II (RR820A, Takara, China) according to the standard protocols. Gene expression values of the control group at 21 and 42 days of the experiment were used for gene expression calibration. The results of qRT-PCR were analyzed by 2^−ΔΔCT^ method [56].

### Determination of inflammatory mediator protein expression levels in the kidney by Western blot

The same samples used for measuring the mRNA expression levels of inflammatory mediator were also used for measuring their corresponding protein expression levels. Kidneys were homogenized in liquid nitrogen with RIPA lysis buffer supplemented with 1 mM phenylmethylsulfonyl fluoride (PMSF). The protein contents of kidney were measured by using the BCA Protein Assay kit. Then the protein samples were separated by sodium dodecyl sulfate-polyacrylamide gel electrophoresis (SDS-PAGE) (10%-15% gels), and protein standards were used as molecular weight marker. After electrophoresis, proteins were transferred to nitrocellulose filter membranes. The membranes were blocked with 5% nonfat-dried milk in phosphate-buffered saline with 0.1% Tween 20 (PBST) for 1 h and then incubated with the primary antibodies overnight at 4°C. The primary antibodies were NF-κB (dilution, 1:5000), IκB (dilution, 1:1000), COX-2 (dilution, 1:1000), TNF-α (dilution, 1:100), IL-1β (dilution, 1:1000), IL-6 (dilution, 1:1000), IL-8 (dilution, 1:800), IL-4 (dilution, 1:500) and IL-10 (dilution, 1:100). After primary antibodies were incubated overnight, the membranes were washed three times with PBST for 10 min and incubated with the biotin-conjugated secondary antibodies for 1 h with gentle shaking, and washed again with PBST. Blots were visualized by ECL^TM^ (Bio-Rad, Hercules, CA, USA) and X-ray film.

### Statistical analysis

The SPSS 17.0 statistical software package programme for windows was used for statistical tests. All results were expressed as mean ± standard deviation. Differences between group means were estimated using one way analysis of variance (ANOVA). A value of *p* < 0.05 or *p* < 0.01 was accepted as statistically significant differences.
